# Emergence of resistance mutations in simian immunodeficiency virus (SIV)-infected rhesus macaques receiving non-suppressive antiretroviral therapy (ART)

**DOI:** 10.1371/journal.pone.0190908

**Published:** 2018-02-21

**Authors:** Benjamin Bruno Policicchio, Paola Sette, Cuiling Xu, George Haret-Richter, Tammy Dunsmore, Ivona Pandrea, Ruy M. Ribeiro, Cristian Apetrei

**Affiliations:** 1 Center for Vaccine Research, University of Pittsburgh, Pittsburgh, Pennsylvania, United States of America; 2 Infectious Diseases and Microbiology, Graduate School of Public Health, University of Pittsburgh, Pennsylvania, United States of America; 3 Pathology, School of Medicine, University of Pittsburgh, Pittsburgh, Pennsylvania, United States of America; 4 Theoretical Biology and Biophysics Group, Los Alamos National Laboratory, Los Alamos, New Mexico, United States of America; 5 Laboratorio de Biomatemática, Faculdade de Medicina, Universidade de Lisbo, Lisboa, Portugal; 6 Microbiology and Molecular Genetics, School of Medicine, University of Pittsburgh, Pittsburgh, Pennsylvania, United States of America; CEA, FRANCE

## Abstract

Two SIVmac251-infected rhesus macaques received tenofovir/emtricitabine with raltegravir intensification. Viral rebound occurred during treatment and sequencing of reverse transcriptase and integrase genes identified multiple resistance mutations. Similar to HIV infection, antiretroviral-resistance mutations may occur in SIV-infected nonhuman primates receiving nonsuppressive ART. As ART administration to nonhuman primates is currently dramatically expanding, fueled by both cure research and the study of HIV-related comorbidities, viral resistance should be factored in the study design and data interpretation.

## Introduction

Due to the reverse-transcriptase (RT) infidelity [1], HIV replication is associated with rapid and frequent development of viral mutations, often leading to the emergence of either noninfectious or less fit strains. Antiretroviral (ARV) drug administration selects for specific mutations allowing the virus to evade the drug(s) [2, 3]. Resistance mutations occur more frequently in ARV-treated HIV-infected subjects with incomplete viral suppression. These aspects are largely ignored for SIV-infected nonhuman primates (NHPs) on ART, even though, until recently, ARV regimens were only partially effective in SIV-infected macaques [4, 5].

SIV variants engineered to harbor known HIV mutations to nucleoside RT inhibitors (NRTIs) and integrase (INT) inhibitors (INTIs) become resistant to these drugs; indicating that HIV and SIV share resistance profiles [6, 7]. Furthermore, SIV may develop *in vitro* mutations against INTI [8], and monotherapy with NRTIs and INTIs may result in emergence of drug-resistant SIV strains *in vivo* [9–11].

Here, we report the development of resistance mutations in SIV-infected rhesus macaques (RMs) in a study of INTI intensification following an initial administration of TFV/FTC. The study of SIV resistance to ARVs is relevant, as ARV administration to SIV-infected NHPs is currently dramatically expanding, fueled by both cure research and research targeting HIV-related comorbidities. For these experiments, it is imperative to design appropriate ART regimens ensuring complete and prolonged viral suppression for the assessment of viral reservoirs and for the control of residual inflammation and immune activation. Preventing the emergence of resistance to ARVs is paramount to such studies.

## Materials and methods

### Ethics statement

RMs were housed and maintained at the University of Pittsburgh, according to the standards of the Association for Assessment and Accreditation of Laboratory Animal Care (AAALAC), and experiments were approved by the University of Pittsburgh Institutional Animal Care and Use Committee (IACUC) (protocol # 16058287). The animals were cared for according to the Guide for the Care and Use of Laboratory Animals and the Animal Welfare Act [12]. Efforts were made to minimize animal suffering: RMs were socially housed together indoors in suspended stainless steel cages, received 12/12 light/dark cycle, were fed twice daily with commercial primate diet, and water was provided *ad libitum*. A variety of environmental enrichment strategies were employed, including providing toys to manipulate and playing entertainment videos in the animal rooms. The animals were observed twice daily for signs of disease or discomfort, any of which were reported to the veterinary staff for evaluation. For sample collection, animals were euthanized with 10 mg/kg ketamine HCl (Park-Davis, Morris Plains, NJ, USA).

### Animals, infections and treatments

Two Indian-origin RMs (*Macaca mulatta*) were analyzed in this study. They were intravenously infected with 300 tissue culture doses (TCID50) of SIVmac251 and were closely clinically monitored throughout infection and treatment.

Starting in chronic infection (250 days postinfection), both RMs received tenofovir (TFV) and emtricitabine (FTC) subcutaneously at 20mg/kg and 40mg/kg once daily, respectively. At 4 weeks, this regimen was intensified with orally-administered raltegravir (RAL) (20mg/kg *bid*). RAL was interrupted after 12 weeks.

### Sampling and sample processing

Blood was collected from all RMs biweekly, with daily blood sampled upon initiation and interruption of RAL for one week. Within one hour of blood collection, plasma was harvested and peripheral blood mononuclear cells (PBMCs) were separated from the blood using lymphocyte separation media (LSM, MPBio, Solon, OH).

### Plasma viral load quantification

We monitored the degree of viral suppression by measuring plasma viral loads (pVLs) on all samples collected. pVLs were tested by using qRT-PCR, using the following primers and probe: SIVmac251F: (5’-GTC TGC GTC ATC TGG TGC ATT C-3’); SIVmac251R: (5’-CAC TAG GTG TCT CTG CAC TAT CTG TTT TG-3’); SIVmac251Probe: (5’-CTT CCT CAG/ZEN/TGT GTT TCA CTT TCT CTT CTG CG/3IABkFQ/-3’) and the conditions described in [4].

### Flow cytometry

Whole blood was stained using a two-step TruCount technique to enumerate the absolute levels of CD4^+^ T cells in the blood, as previously described [13]. The following fluorescently-labeled antibodies were using for staining blood (all antibodies from BD Biosciences, San Jose, CA, USA): CD4 (APC), CD8 (PE-CF594), CD3 (V450), CD45 (PerCP), Ki-67 (PE). Ki-67 was stained by first fixing and permeabilizing cells prior to staining. Flow cytometry acquisitions were performed on an LSR-II flow cytometer (BS Biosciences).

### Assessment of the occurrence of resistance mutations

The RT and INT genes of the SIVmac variants circulating in plasma samples collected prior to initiation of ART [0 weeks post-treatment (wpt)], as well as 2, 8, 12 wpt and finally, after cessation of RAL (20 wpt) were sequenced to determine the presence of resistance mutations. The following primers were used for PCR and sequencing: RT outer primers RT3 (5’-GTT GCA TTA AGA GAA ATC TGT GAA AAG ATG G-3’) and RT5 (5’-CCA GGT CTC TCT TTG TGG CAA CTC-3’) and inner primers RT6 (5’-CCA ATC CAT ACA ACA CCC CCA C-3’) and RT7 (5’-CAA CTT CCA TTT TGT CGG CCA C-3’); INT outer primers INTF1 (5’-CAT GGG CAG GTA AAT TCA GAT C-3’) and INTR1 (5’-TAT CCC CTA TTC CTC CCC TTC-3’), and nested primers INTF2 (5’-TAG GGA CTT GGC AAA TGG AYT G-3’) and INTR2 (5’-CTG AAT TTG CTT GTT CCC TGA TTC-3’). These partial gene sequences correspond to specific regions within the RT (566 bp fragment, between N54 and L241) and INT (342 bp fragment, between G59 and S171) known to encompass common resistance mutations. PCR conditions were as follows: RT outer: initial denaturation of 5 minutes at 95°C; 30 cycles of 1 min at 95°C, 30 sec at 54°C, 45 sec at 68°C; and a final elongation of 5 min at 68°C. RT inner: initial denaturation of 5 min at 95°C; 40 cycles of 1 min at 95°C, 30 sec at 51°C, 45 sec at 68°C; and a final elongation of 5 min at 68°C. INT outer: initial denaturation of 5 min at 95°C; 30 cycles of 1 min at 95°C, 30 sec at 50°C, 30 sec at 68°C, 30 sec; and a final elongation of 5 min at 68°C. INT inner: initial denaturation of 5 min at 95°C; 40 cycles of 1 min at 95°C, 30 sec at 51°C, 30 sec at 68°C; and a final elongation of 5 min at 68°C. PCR products were gel-purified and submitted to conventional sequencing using the nested primers.

The RT and INT sequences were aligned to the SIVmac251 isolate Mm251 (GenBank: M19499.1) using Sequencher software and the emergence of resistance mutations was assessed based on the Stanford HIV Drug Resistance Database (https://hivdb.stanford.edu).

### Nucleotide sequence accession numbers

The nucleotide sequences of the RT and INT from the RMs included in these studies were deposited in the GeneBank (accession numbers: MG686471-MG686486).

## Results and discussion

**Fig 1:**
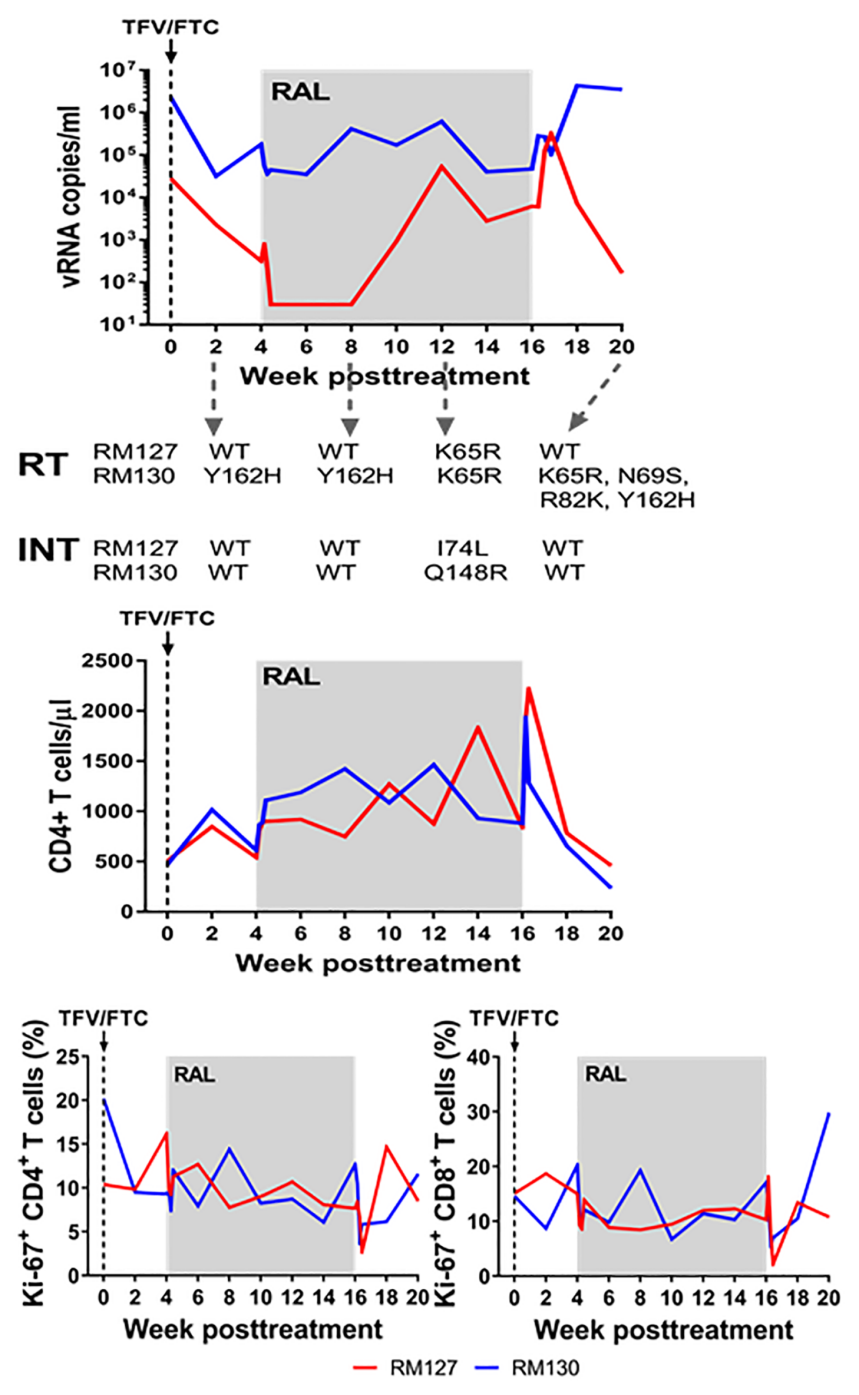
Administration of nonsuppressive ART to two SIVmac251-infected rhesus macaques resulted in development of resistance mutations and treatment failure. (a) Plasma viral load levels following treatment with TFV/FTC followed by intensification with RAL four weeks later. Reverse transcriptase (RT) and integrase (INT) regions of virus amplified from plasma at select points post-treatment listed in the figure were sequenced to determine the presence or absence of ART-resistance mutations. In the absence of detected resistance mutations at the selected time points, those strains are considered wild type. (b) Peripheral CD4^+^ T cell counts during the follow-up; (c) Changes in the levels of circulating CD4^+^ and CD8^+^ T cell activation during the follow-up, as documented by the frequency of Ki-67 expression.

Upon initiation of the TFV/FTC treatment, a ~1.5 log decrease was achieved within the first two weeks in RM130, followed by a 0.7 log rebound in pVL. In RM127, a ~2.5 log decrease in pVL occurred, which was maintained through week 4. Following RAL intensification, at 4 weeks, a ~0.5 log decrease in pVLs occurred in RM130, which was maintained for 2 weeks, but was rapidly followed by a robust pVL rebound throughout the follow-up. Conversely, in RM127, RAL intensification further suppressed the virus, with pVLs below the limit of detection for at least four weeks. However, at week 8, pVLs rebounded by ~3 log and remained high throughout RAL treatment. Robust pVL rebounds were observed in both animals (~2 log in RM130 and ~1.5 log in RM127) after RAL discontinuation (Fig 1a).

In both animals, CD4^+^ T cells recovered slightly after initiation of NRTIs, with a further recovery following RAL intensification, which was reversed after RAL cessation (Fig 1b), although there was an initial unexplained spike in the number of CD4^+^ T cells. The frequency of CD4^+^ and CD8^+^ T cells expressing Ki-67 remained stable during RAL intensification, and increased after RAL cessation (Fig 1c).

Such patterns of viral replication under ART being suggestive of resistance mutations, we investigated whether this incompletely suppressive treatment resulted in the emergence of drug-resistant strains. Serial sequence analyses demonstrated that major and minor mutations emerged in both genes in both animals (Fig 1a). Thus, sequence analyses showed that, in RM130, major drug resistance mutations occurred at the same time point (12 wpt) in both INT (Q148R) and RT (K65R). Conversely, in RM127, only one major mutation was observed to occur only in the RT at one time point (K65R, 12 wpt), while only one minor mutation was selected in the INT gene at the same time point (I74L, 12 wpt). In humans, occurrence of these mutations was reported to be associated with treatment failure: K65R results in a 3.3–3.6-fold increase in resistance to TFV and Q148R results in a 44-46-fold increase in resistance to RAL [14].

Multiple minor mutations were observed at several time points (2, 8, and 20 wpt) in RM130 RT, with no minor mutations occurring in RM127 (Fig 1). Interestingly, in spite of cessation of RAL treatment, VLs decreased in RM127 at 20 wpt. This may be due to an boost of cell-mediated immune responses against the rebounding resistant virus, as previously reported [11]. Further, the reversion of the K65R mutation to wild type at 20 wpt may explain the observed decrease in VL in RM127.

The mutation M184V/I is a known, frequently-selected major mutation against FTC. The viral strains harboring this mutation have an increased susceptibility to TFV [15, 16]. In our study, none of the sequences from any of the RMs at any time points yielded this major mutation, in spite of the inclusion of FTC in the regimen leading to incomplete SIV suppression. The reasons for the lack of selection of M184V/I may be multiple: (i) first, it is possible that M184V/I never occurred under the combination of TFV and FTC; (ii) second, it is possible that M184V/I occurred, but, as was previously reported [17], it increased viral susceptibility to TFV, thus drastically reducing the fraction of the viruses carrying M184V/I in the virus quasispecies, limiting the probability of detection by Sanger sequencing; (iii) M184V/I occurred but, as was previously reported [18], the fitness of the virus carrying it was drastically altered, resulting again in a frequency of the mutated strains below the limits of detection by Sanger sequencing. Analysis through deep sequencing might thus permit the detection of such minor variants in the quasispecies.

Our analysis documented the accumulation of ART resistance mutations as the cause of treatment failure in two analyzed RMs. To our knowledge, it is very uncommon to find SIV resistance to ARV *in vivo* in RMs on combination therapy, probably due to the fact that in the past, the duration of treatment was relatively limited, with the treatment given just for the duration of the *in vivo* experiments and for a much shorter length of time than in HIV-infected subjects [19, 20]. Previous studies reporting that SIV may share similar resistance profiles to HIV and can develop resistance mutations against NRTI or INTI were performed in monotherapy [6–11].

Clinical trials conducted during the last two decades were plagued by HIV resistance to ART [21], but emergence of viral strains carrying resistance mutations to ARVs tends to be ignored when designing and performing NHP studies involving treatments. This is particularly true when the experiments include ARVs administered orally, as in such experimental environments it is very difficult to ensure adherence to treatment in NHPs. The emerging fields of HIV cure and comorbidities combined with new highly-effective suppressive ART regimens for macaques have increased the use of the ART-treated macaque model [22]. However, inappropriate drug regimens or incomplete administrations will not only impact our ability to study the viral reservoirs or HIV-related comorbidities, but will also increase the likelihood of the emergence of SIV resistance to ARVs. The addition of an INTI to the drug regimen effectively reduces viremia [22, 23], suggesting the importance of using first-line ART regimens used in humans for insuring robust and persisted control of the virus. As both cure and comorbidity research require prolonged viral suppression to ART, resistance to ARVs need to be considered as a potentially critical limitation factor in the rapidly emerging field of modeling ART in NHPs, particularly when nonsuppressive ART regimens are used.
